# Operative Treatment in Chest Surgery

**Published:** 1918-08

**Authors:** A. L. Lockwood


**Early Operative Treatment in Chest Surgery.** By Major A. L. Lockwood, R. A. M. C.

The speaker stated that complete intrathoracic operations, such as he was to describe, should be undertaken only in cases likely to prove fatal. There is danger of too general a use of this technic.

Until 1916, expectant treatment of chest wounds was considered sufficient, and even the excision of the parietal wound was not undertaken unless it was badly infected. It was soon recognized, however, that chest wounds involving the diaphragm and abdomen demanded immediate operation, but that they were not necessarily fatal. The repair of the diaphragm was observed to be more urgent than that of any hollow or solid viscus in the abdomen. In general operation should be undertaken as early as possihle.

The procedures finally evolved were : 1. Excision of parietal wound and removal of fragments and (when possible) closing of pleura. No attempt was made to clear the hemothorax or to follow the missile or to repair the thoracic content.

2. In 1916 repair of the diaphragm by the abdominal route became a routine procedure.

3. When it was found that even without sepsis death resulted usually from badly comminuted parietal fractures, it was made a routine practice to operate immediately all " stove in " chests.

4. The treatment of thoracic wounds of the diaphragm by the thoracic route.

5. In the case of sucking wounds (traumatopnea) which had previously proved invariably fatal, it was found practicable to excise the parietal wound, clear out the hemothorax, and close the chest wall.

Bad cases, especially those with sucking wounds should not be removed from advanced dressing stations until partly rallied. For traumatopnea, the skin should be sutured with silkworm gut through a plug of gauze. As soon as the leakage is stopped, great improvement is at once apparent and the lung tends to expand.

For resuscitation; the patient is placed, if possible, with the injured side dependent. Continuous rectal administration of soda bicarbonate and glucose (5 per cent. each in water) is started. Intravenous soda bicarbonate (2 per cent) may also be given. In severe cases blood transfusion (600 to 800 cc.) is administered.

Hot drinks by mouth, but no stimulants whatever, are given. Sleep should be induced by every possible means. Omnopon or morphin (preferably the former) may be used. Dyspnea from hemothorax or pneumothorax should be relieved before operation by aspirating.

The most perfect operative conditions are essential to success. If operation is undertaken in these severe cases :

1. Operate as soon as patient's condition allows.
2. Operate when *injury to the diaphragm is suspected.*
3. Operate when evacuation will not be necessary.
4. Operate in all cases of open pneumothorax.
5. Operate on all badly " stove in " chests, even if there is no external wound.
6. Operate in all cases where a large missile has traversed the pleural cavity, wherever it may be lodged.
7. Operate on all badly infected wounds, even if the missile is not retained.

Most complete and constant X-ray investigation is necessary. Full size stereoscopic plates should be taken. Early operation on cases showing signs of bronchopneumonia on the uninjured side must not be undertaken lightly and only with local anesthesia.

The speaker insisted upon the exacting nature of operative technic in dealing with such cases.

A separate theater heated uniformly to 80° F. is reserved for this work. Perfect asepsis is maintained.

If the patient is not placed with the wounded side dependent, the lung should be grasped with forceps of the Collin pattern; and the displacement of the mediastinum corrected.

The skin is prepared with a 3 per cent. picric acid in spirits. Paravertebral anesthesia is administered two or three spaces above and below the wound. A local infiltration at some distance from the wound is employed.

Novocain 5 per cent, and potassium sulphate 0.25 per cent in normal saline, prepared fresh and repeatedly autoclaved, is injected with a Gray's syringe (10 minims of adrenalin per ounce are added just before use). Gas and oxygen should be available for administration while the hand is inside the chest or when the patient is restless.

The most serious cases may be operated on with a light nitrous oxide analgesia. Local anesthesia combined with gas and oxygen is the best means of preventing shock in extended operations. Neither ether nor chloroform should be used in chest surgery.

When the position of the wound permits, resection of the fourth rib from the mid-clavicular to the posterior axillary line furnishes the easiest access to the thoracic cavity. Resection of the rib should be wide enough to allow careful inspection of the cavity. Doyen's periosteal elevator and costotome are the instruments best suited for the resection of the rib. Tuffier's retractor is useful.

The thoracic cavity must be mopped out with gauze wrung out of hot saline carried on a long curved forceps of the Ochsner pattern.

When a missile has pierced the diaphragm and entered the liver, the diaphragm must be excised widely enough to expose the tract in the liver, and the missile removed. The liver tract should be cleansed with Volkmann's spoon followed by swabs wrung dry out of saline and ether.

If oozing occurs, deep catgut sutures should be inserted with a blunt needle. Mattress sutures suffice to close the diaphragm, except when it is stripped from its parietal attachment. The diaphragm, however, need not be sutured to the chest wall.

It is wise to deal with abdominal wounds after closure of the chest.

Partial lobectomy may be necessary depending on the degree of laceration of the lung. Total lobectomy and excision of both middle and lower lobes of the right lung have been necessary for acute malignant gas gangrene, but it has not saved the patient's life. An open bronchus is rarely found at operation, Crushing and ligaturing with catgut is sufficient.

The visceral surface of the lung should in all cases be approximated, thus mechanically retarding effusion from the damaged lung, and lessening the tendency for infective conditions to light up in the lung substance itself. Hemorrhage from the lung need not be feared.

All foreign bodies and blood clots should be removed from the thoracic cavity. The toilet of the pleura can better be performed by sponging (as in the case of the peritoneum) than by washing out. The last step before closing off the pleural cavity is to sweep round the chest wall, lung, mediastinum, and diaphragm systematically with swabs wrung dry out of hot saline, and, finally with a swab wrung dry out of warmed ether.

The chest should always be closed, unless there is extensive gas gangrene of the lung tissue itself adherent to the chest wall.

Time should not be wasted in attempting to repair the parietal pleura in extensive wounds, as it can rarely be done; such pleura as remains can be caught up with the muscle sutures.

The chest must be hermetically closed with the first layer of muscles, otherwise pocketing will occur, pleural effusion accumulate, the incision break down, and the operation fail. From the time the pleura is opened until it is closed, when the hands of the operator are not actually in the chest, the hole in the pleura should be covered by thick lint wrung dry out of hot saline. This closure is important, even if only for a moment at a time.

Careful approximation of the skin edges is necessary to ensure early and absolute primary union.

A wide gauze dressing with mastisol or " aeroplane dope " reduces the tension on the sutures and binds the layers of the chest wall so as to prevent oozing and pocketing. A binder made of 7 in. adhesive plaster (tying over the dressing) is valuable to retain the dressing, as well as to relax tension on the sutures and leave the sound side of the chest free for expansion; the latter is extremely important.

On completion of the operation, the patient should at once be supported in a semi-recumbent position inclined to the injured side.

The two-stage operation is indicated in the type of case with the entrance and exit wounds far apart — front and back or lateral — where gross lesion of the bone, or extensive destruction of the tissues, necessitates an extensive operation of both wounds. In such a case, enter the chest through the wound which allows freest access to the pleural cavity and to the part probably damaged. After carrying out the operation as outlined above, leave the patient on the table in a comfortable position, surrounded by hot-water bottles. Give intravenous sodium bicarbonate or blood transfusion as required, administer sodium bicarbonate and glucose 8 oz. per rectum. Half an ampoule of omnopon should be given if the patient is at all restless. After one or two hours, deal with the other wound. For the second stage, a further para-vertebral anesthesia is frequently not required — merely local infiltration about the site of the incision.

Injuries of the heart or pericardium can be best dealt with by a parasternal flap of the fourth and fifth, or the fifth and sixth, costal cartilages depending on the probable site of the lesion (the divided cartilages unite rapidly); and this route, in addition, gives free access to the pleural cavity.

Where the missile has passed across the pleural cavity and lodged in the mediastinum, especially high up, it is wiser to enter the mediastinum through the sternum. The missile should be removed, its bed and track thoroughly cleaned, and the pleural opening closed to prevent any leakage from the mediastinum into the pleural cavity. This serves a double purpose — it obliterates a pocket in which pleural effusion might accumulate, and shuts off from the pleural cavity a source of reinfection. It is difficult to deal with the mediastinum through the usual costal incision.

Gross lesions of the parieties under the scapula are always difficult to reach. It is possible to deal with such wounds from either vertebral or axillary border.

Above all, speed and absolute asepsis are essential to success. The operation must begin with *excisio in toto* of the wound and end with hermetical sealing of the thorax.

In no class of surgery is team work so essential to success. The surgeon, physician, radiographer, and anesthetist should work hand in hand. The theater nurse should be particularly quick and methodical, knowing each step in the operation; and avoiding delay by having everything prepared in advance and at hand. A post-operative nurse, who has had long experience in the nursing of these cases, is a most important member of the team.

